# Prostate cancer mortality and costs of prostate surgical procedures in the Brazilian public health system

**DOI:** 10.1590/S1677-5538.IBJU.2021.0781

**Published:** 2022-01-28

**Authors:** Allan Saj Porcacchia, Gabriel Natan Pires, Valdemar Ortiz, Monica Levy Andersen, Sergio Tufik

**Affiliations:** 1 Universidade Federal de São Paulo - UNIFESP Departamento de Psicobiologia São Paulo SP Brasil Departamento de Psicobiologia, Universidade Federal de São Paulo - UNIFESP, São Paulo, SP, Brasil; 2 Universidade Federal de São Paulo - UNIFESP Departamento de Cirurgia São Paulo SP Brasil Departamento de Cirurgia, Universidade Federal de São Paulo - UNIFESP, São Paulo, SP, Brasil

## INTRODUCTION

Prostate cancer (PCa) is the most frequent type of cancer in the male population in 112 countries. PCa represents 14.1% of the incidence and 6.8% of the mortality by cancer worldwide, which represents 1.4 million new cases and 375.000 deaths in 2020 (1). In the same year, the age-standardized incidence rate of PCa was 65.5, whereas the mortality was 13.6 per 100.000 individuals in South America (1). According to the Instituto Nacional de Câncer José Alencar Gomes da Silva (INCA), one of the main Brazilian cancer institutes, PCa accounts for 29.2% of all cancers in males in Brazil (2). This represents 65.840 new cases of PCa in each year in the period 2020-2022 (2).

The etiology of PCa is still poorly understood and the most well-known risk factors are older age, genetic mutations, and a family history of this type of cancer (3). Smoking, excessive body weight and diet are some environmental factors that may be related to this cancer, but further studies are still necessary to confirm this (1, 3).

The guidelines for managing localized PCa depend on the severity of the tumor and on the risk group to which the patient belongs, according to the American Society of Clinical Oncology (4). The main approaches are surgical procedures (prostatectomies), radiotherapy, and androgen deprivation therapies. They can be applied alone or in combination. For low and very low risk localized prostate tumors in patients who have a high probability of progression, physicians may offer surgical procedures as a definitive treatment. In cases of intermediate and high risk localized PCa, prostatectomy or radiotherapy with androgen deprivation therapy should be recommended (4).

In Brazil, citizens have access to the Sistema Único de Saúde (SUS, the Brazilian Unified National Health System) which is responsible for undertaking preventive exams and treating numerous disorders and diseases, including cancer. Information regarding the number of hospitalizations, procedures, costs, number of deaths, mortality rates and other factors related to the treatment of PCa by SUS are available in the public database DATASUS. Furthermore, INCA is responsible for the Atlas On-line da Mortalidade (here referred to as the Online Atlas of Cancer Mortality), a database presenting information specifically on deaths and mortality rates related to cancer in Brazil (5). The main focus of this study is to present a description of data concerning PCa mortality, diagnosed cases, and the costs of prostate surgical procedures in the SUS based on these two public databases.

## MATERIAL AND METHODS

### Databases search

Two publicly available databases which contain data on the Brazilian public health system were used as the primary sources for this study: the Online Atlas of Cancer Mortality of INCA and the DATASUS. The search collected information regarding mortality due to malignant neoplasm of the prostate (ICD-10-CM C61) between 1994 and 2019 from the Online Atlas of Cancer Mortality. The following data were gathered for each year: the number of deaths, the crude mortality rate of PCa, age-specific mortality rates of PCa, the age-standardized mortality rate for the world population mortality rate of PCa (considering the standard world population described by Doll et al. (6)), and the age-standardized mortality rate for the Brazilian population (considering Brazil's standard population of 2010 (6, 7)). Age standardization was applied in order to obtain results that are independent of the effects of age. The age-standardized mortality rate for the world population mortality rate of PCa will be referred to here only as “age-standardized mortality rate” and will be used to estimate the mortality trends of this type of cancer in the country. Brazil comprises five geographic regions -the North, Northeast, Central-West, Southeast, and South. The number of deaths according to regions and age were also included.

The search of the DATASUS database collected data regarding the number of PCa diagnoses, as well as the number and costs of hospitalizations for prostate surgical procedures between 2008 and 2020. More specifically, these procedures encompassed suprapubic prostatectomy, and radical prostate-vesiculectomy, which are referred to as prostatectomy in oncology, and radical prostate-vesiculectomy in oncology in the databases when used to treat PCa.

Searches in both databases were performed in April 2021. Submission of this work to the institutions Research Ethics Committee was not necessary, as data from public domain databases were used.

Trends of mortality rates in cancer epidemiology can be estimated by the annual percent change (APC) within a certain period. A positive value represents a rise in the mortality rate, whereas a negative one indicates a decline. The joinpoint regression method (8) was used to analyze age-standardized mortality rate PCa trends in the period 1994-2019 (95% confidence interval-CI). The value of the average annual percent change (AAPC) is a summary of APCs over a period of several years. The Joinpoint Regression Program (Version 4.8. 0.1; Statistical Methodology and Applications Branch, Surveillance Research Program, National Cancer Institute) was used for this analysis (9). The standard controls of the software were used, and constant variance/homoscedasticity was assumed in respect of errors.

## COMMENTS

The number of deaths due to PCa increased in the Brazilian male population during the period 1994-2019, going from 5.256 in 1994 to 15.576 in 2019. Consequently, crude mortality rate and age-standardized mortality rates showed a gradual rise over the years (Table-1).

**Table 1:** Deaths and mortality rates of PCa per 100.000 individuals 1994-2019 in Brazil.

Year	Deaths	Crude mortality rate (%)	Age-standardized mortality rate for world population (%)	Age-standardized mortality rate for Brazilian population (%)
1994	5.256	6.93	10.84	12.76
1995	5.542	7.20	11.28	13.22
1996	6.067	7.83	10.96	12.74
1997	6.652	8.45	11.83	13.88
1998	7.144	8.95	12.57	14.79
1999	7.223	8.93	12.57	14.83
2000	7.490	8.96	11.39	13.35
2001	8.033	9.20	12.24	14.48
2002	8.389	9.48	12.44	14.75
2003	8.977	10.02	12.95	15.37
2004	9.590	10.57	13.41	15.98
2005	10.214	11.13	13.81	16.55
2006	11.007	11.86	14.35	17.27
2007	11.478	12.23	14.42	17.34
2008	12.121	12.78	14.61	17.67
2009	12.274	12.82	14.25	17.24
2010	12.778	13.68	13.25	15.98
2011	13.129	13.45	14.10	17.14
2012	13.354	13.56	13.84	16.78
2013	13.772	13.86	13.73	16.70
2014	14.161	14.14	13.59	16.53
2015	14.484	14.35	13.38	16.25
2016	14.926	14.78	13.79	16.74
2017	15.391	15.25	14.24	17.25
2018	15.576	15.43	14.41	17.47
2019	15.983	15.83	14.79	17.92

* Adjusted by age according to world standard population per 100.000 individuals as described by Doll et al. (6).

# Adjusted by age according to Brazilian population of 2010 per 100.000 individuals.

Data from Online Atlas of Cancer Mortality from INCA (available at. https://mortalidade.inca.gov.br/MortalidadeWeb.

**Figure 1:**
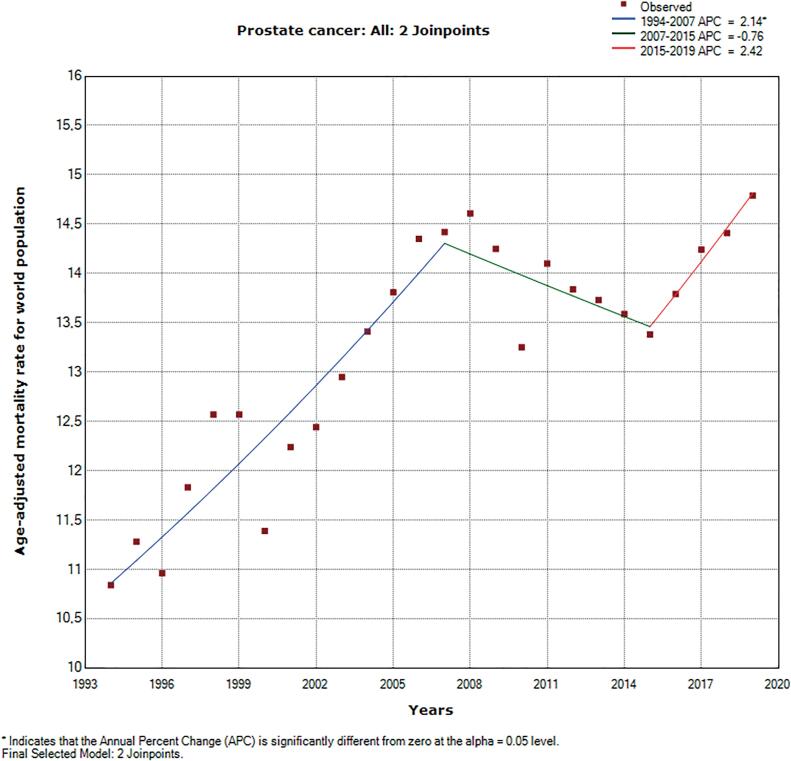
Age-standardized PCa mortality rates in Brazil 1994-2019. The joinpoint regression conducted resulted in a positive AAPC of 1.3% (95% CI, 0.6-1.9; p <0.05) considering the age-standardized mortality rate of PCa in Brazil between 1994 and 2019.

In order to evaluate trends in PCa mortality over the years in Brazil, the joinpoint regression method was used with the age-standardized mortality rates of PCa between 1994 and 2019. The algorithm identified two joinpoints in which the trends of PCa mortality of the Brazilian male population have changed significantly, located in the years 2007 and 2015 (Figure-1). For the first period (1994-2007), the APC was 2.14% (95% CI, 1.6-2.7), indicating increasing mortality. For the second period (2007-2015), the APC was −0.76% (95% CI, −2.1-0.6). The last APC calculated was 2.42% (95% CI, −0.8-5.7) and refers to the period 2015-2019. Considering the CI of the 2^nd^ and 3^rd^ APCs, it is not possible to affirm whether the trends of age-standardized mortality rate of PCa were increasing or decreasing during the respective periods. The AAPC for the whole period (1994-2019) was 1.3% (95% CI, 0.6-1.9; p <0.05), which shows an increasing trend in the age-standardized PCa mortality rate during this period.

**Figure 2:**
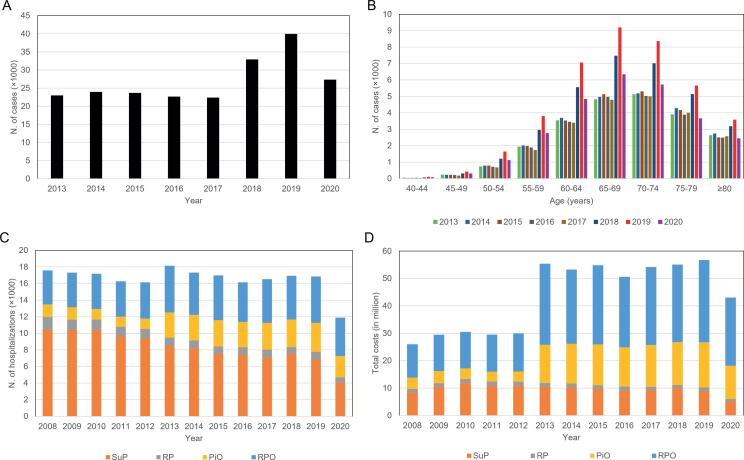
A) Number of PCa cases diagnosed in SUS in Brazil 2013-2020. B) Number of PCa cases diagnosed in SUS in Brazil by age 2013-2020. C) Number of hospitalizations in SUS Brazil for prostate surgical procedures 2008-2020. D) Total costs (in BRL) of hospitalizations for prostate surgical procedures in SUS Brazil 2008-2020. Procedures - SuP: suprapubic prostatectomy; RP: radical prostate-vesiculectomy; PiO: prostatectomy in oncology; RPO: radical prostate-vesiculectomy in oncology. Data from the database DATASUS (http://www2.datasus.gov.br/DATASUS/index.php).

Data from the DATASUS database showed that the number of PCa diagnoses in SUS remained relatively constant between 2013 and 2017: the lowest number was 22.396 in 2017, and the highest was 23.942 in 2014. In 2018 and 2019, the number of PCa diagnoses increased from 32.930 to 39.953, but fell to 27.358 in 2020 (Figure-2A).

In the period 2013-2020, the number of PCa cases in SUS varied according to the age-range (Figure-2B). The yearly total of diagnoses was progressively higher in the 55-69 age group. A slightly gradual decrease was observed in the 70-74, 75-79, and ≥80 age-groups. Despite the growth in the total amount of diagnoses between 2017 and 2019 within the age-groups over 50 years old, a decrease occurred immediately after in 2020.

An analysis of the number of hospitalizations for prostate surgical procedures reveals that it remained relatively similar between 2008 and 2019 (mean±standard deviation; 16.944±582.6 per year) (Figure-2C). In 2020, this number decreased to 11.875, which is 5.609 less than the mean of the previous years.

Between 2008 and 2012, the total costs of hospitalizations for prostate surgical procedures in SUS remained below 31 million BRL per year, and then increased to more than 50 million BRL in subsequent years, except for 2020. Radical prostate-vesiculectomy in oncology was responsible for the greatest costs in respect of prostate surgical procedures in each year in the whole period (Figure-2D). Although radical prostate-vesiculectomy in oncology represented the highest cost in all years of the period, since 2013 the total cost of this procedure per year has practically doubled (27.8 million±1.7 million BRL; 2013-2020 period) compared to the period 2008-2012 (13.2 million±0.59 million BRL).

As in most countries, PCa is the most incident type of cancer in the male population of Brazil, excluding non-melanoma skin cancer (1, 2). In the period 1994-2019, the age-standardized PCa mortality rate increased in Brazil, with an AAPC value of 1.3% for the period. When considering the increase in the age-standardized PCa mortality rate in Brazil, a factor that should be considered is the aging of the population. Older adults are not homogeneously distributed throughout Brazil. The number of inhabitants over 60 years old is greater in the Southeast and Northeast regions, representing 46.4% and 25.5% of the total number of over 60s in Brazil, respectively, while the population of these two areas combined represents 70% of the total population (10). Both regions also show the highest number of deaths due to PCa. Although 70% of the Brazilian male population resides in the Southeast and Northeast regions (10) and these present the highest concentration of people over 60 years of age, the southern region of Brazil has the highest number of deaths from PCa per 100.000 individuals. This scenario suggests that male inhabitants of the South region may be under the influence of some risk factor (environmental or genetic variant) related to PCa and/or that these individuals have greater access to health services, which enables the correct notification of deaths due PCa.

The incidence and mortality rates of PCa vary widely across the regions of a country, and between different countries (11). In addition to aging, exposure to risk factors, and lack of access to preventive medicine, clinical exams, or treatments are the key components that contribute to the higher incidence and mortality rates (1, 11). In many developing countries, there is still an increase in the incidence and mortality rates of PCa, and these may be related to more aggressive tumor types or inadequate access to health care systems (12). This last factor may not be the case of Brazil, as the growth in PCa incidence took place at the same time as access to PCa screening/diagnosis and medical care were increasing (13). Between 1999 and 2007, the number of prostatic specific antigen (PSA) tests - the main biomarker of PCa - performed in SUS increased by 573.3%, even though the Ministry of Health and INCA did not recommend PSA screening (14). The Sociedade Brasileira de Urologia (SBU, the Brazilian Urology Society) did support screening for men aged over 50 or over 45 with high risk (13). A limitation of the present study is that we can only speculate in relation to the potential factors involved in the growth trend in mortality and incidence rates of PCa. Further studies are required to evaluate this in more detail, including the evaluation of population genetic variance of target genes as a risk factor.

Although there was an increase in the number of PCa diagnoses in SUS between 2017 and 2019, this decreased in 2020. This apparent decrease observed in all ages over 50 may not be an actual decrease but may be due to the fact that COVID-19 caused a significant drop in face-to-face consultations and, therefore, diagnoses. There was also a drop in the number of hospitalizations for surgical prostate procedures in SUS in 2020 (15). However, it is important to remember that these measures of physical distancing were essential to prevent deaths and conserve the limited supplies for professionals working in the frontline of the pandemic (16). There may also have been a possible delay in notifications in respect of these data due to the disruption caused by COVID-19, although this cannot yet be confirmed.

The search for procedures related to the prostate on the DATASUS database resulted only in data in respect of hospitalizations and costs regarding the surgical approaches: suprapubic prostatectomy, radical prostate-vesiculectomy, prostatectomy in oncology, and radical prostate-vesiculectomy in oncology. The data regarding the DATASUS database refer only to information and records in the public health system in Brazil, the SUS. There is no centralized data regarding the number of PCa diagnoses in the private sector and the related surgical costs. Future studies comparing the public and private sectors should be considered.

The costs of the prostate surgical procedures presented here are a part of the total costs related to the disease (diagnosis, treatments, palliative care, follow-up, potential side effects, and human resources of health professionals), and indirect costs, relating to factors, such as the time and productivity of the patients (17). The early detection of tumors is essential for a good prognosis following treatment and to avoid the costs of more complex approaches (17). In this respect, the use of the PSA test for screening and detection of PCa has been widely discussed, and recommendations in relation to its use vary among different countries, scientific societies, and surveillance institutes (13, 18, 19). However, the positive impact of the PSA test in respect of the early diagnosis and treatment of PCa over the last four decades (19) must be considered in further discussions regarding this issue.

## CONCLUSIONS

PCa, similar to several other types of cancer, is an age-related disease. Given the rapid aging trend of the Brazilian population, the number of PCa cases may increase, as other types of cancer and conditions associated with age. SUS and private health systems must be aware of this scenario. Investments need to be made in order to improve epidemiologic cancer surveillance programs, and increase clinical and hospital resources to provide the most effective and modern treatments for the population. The impact of the COVID-19 pandemic should be considered in data regarding PCa from 2020 forward, and in future epidemiological studies of this disease.
